# Disease severity among hospitalized children during the COVID-19 pandemic in Israel

**DOI:** 10.1007/s10096-026-05485-6

**Published:** 2026-04-09

**Authors:** Ilan Livne, Yair Goldberg, Amit Huppert, Michal Stein, Shirley Shapiro Ben David

**Affiliations:** 1 Neaman Institute for National Policy Research, Technion – Israel Institute of Technology, Haifa, Israel; 2 Technion - Israel Institute of Technology, Haifa, Israel; 3 Data & Analytics, Sheba Medical Center, Ramat Gan, Israel; 4 Department of Epidemiology and Preventive Medicine, the Gray Faculty of Medical & Health Sciences, Tel Aviv University, Tel Aviv-Yafo , Israel; 5 Pediatric Infectious Disease Unit, The Edmond and Lily Safra Children’s Hospital, Chaim Sheba Medical Center, Ramat Gan, Israel; 6 Faculty of Medical and Health Sciences, Tel Aviv University, Tel Aviv, Israel; 7 Maccabi Healthcare Systems, Division of Health, Infectious Disease Unit, Tel Aviv, Israel; 8 The Gertner Institute for Epidemiology & Health Policy Research, Sheba Medical Center, Ramat Gan, Israel

**Keywords:** COVID-19, Pediatric hospitalization, Disease severity, Risk factors, Public health

## Abstract

**Introduction:**

COVID-19 typically results in mild illness among children, but severe cases do occur, leading to hospitalizations and critical outcomes such as respiratory failure. High-quality surveillance of COVID-19 presents a unique opportunity to understand risk factors and changing patterns of severity across different pandemic waves.

**Aim:**

We aimed to assess the burden of COVID-19-related hospitalizations among children in Israel, identify key risk factors for moderate-to-severe disease among hospitalized children, and examine trends in disease severity across different phases of the pandemic.

**Methods:**

We conducted a retrospective cohort study of all children aged 0–18 years hospitalized with COVID-19 within Maccabi Healthcare Services from March 2020 to November 2023. Data on comorbidities, age, vaccination status, and other covariates were extracted from electronic health records. Disease severity was classified as mild or moderate-to-severe. Logistic regression was used to estimate adjusted odds ratios for moderate-to-severe illness.

**Results:**

In a pediatric population of approximately 770,000 children during a 3.5-year study period, 935 COVID-19–related hospitalizations were identified (approximately 35 hospitalizations per 100,000 children per year), and severe outcomes were uncommon. Among hospitalized children, most experienced mild disease (822/935), with 57 moderate cases and 56 severe or critical cases or deaths. Overall, 12.1% of hospitalizations progressed to moderate-to-severe illness. Disease severity was strongly associated with comorbidity burden (OR = 1.98 for one condition; OR = 5.88 for two or more conditions). Lack of vaccination was also associated with higher odds of severe outcomes (OR = 5.94), although estimates were imprecise due to sparse events.

**Conclusions:**

Over more than three and a half years of follow-up, COVID-19 was characterized by a minimal burden of severe disease in the pediatric population. Even among hospitalized children, clinically meaningful risk was observed in only a small subset, emphasizing the importance of focused risk stratification rather than broad generalized concern. Overall, these findings highlight the predominantly mild course of COVID-19 in children across successive pandemic periods.

**Supplementary Information:**

The online version contains supplementary material available at 10.1007/s10096-026-05485-6.

## Introduction

Coronavirus disease 2019 (COVID-19) generally causes mild illness in children, with far lower rates of hospitalization and mortality compared to adults [1]. Nevertheless, severe, critical cases and death do occur in the pediatric population, including pneumonia with respiratory failure and multisystem inflammatory syndrome in children (MIS-C). In Israel, by October 2021, prior to the Omicron BA.1–2 wave, 2,660 children under 18 had been hospitalized with COVID-19, and 398 of these cases were classified as severe [2]. This demonstrates that while the risk of severe disease is low on a population level, a substantial number of children have experienced severe outcomes. Identifying which children are at greatest risk is of interest for understanding future outbreaks with a wide infection rate and low hospitalization rates.

Early in the pandemic, pediatric COVID-19 hospitalizations were relatively infrequent, in part due to widespread implementation of non-pharmaceutical interventions (NPIs) that reduced infections, and cases were often concentrated among children with underlying medical vulnerabilities. As the virus evolved and more children were infected, a broader spectrum of pediatric hospitalizations was observed, especially during surges of more contagious variants. It is unclear whether the risk profile for severe disease in children changed throughout the pandemic (for example, with the emergence of the Omicron variant in late 2021). Evidence from other settings suggests that the burden of severe pediatric cases shifted towards younger ages during the Omicron BA.1–2 wave, as older adolescents benefited from vaccination and prior exposures [1]. There is also evidence that the intrinsic virulence of SARS-CoV-2 in children became lower with Omicron BA.1–2; one U.S. cohort study found that while the Omicron wave produced the highest volume of pediatric COVID-19 admissions, the rate of severe disease was significantly lower than in the Alpha and Delta variant waves [3]. Meta-analyses further indicate that children consistently experienced lower risks of hospitalization and severe outcomes than older age groups across major variants [4].

Several studies have consistently reported that children with chronic comorbidities are at higher risk of severe COVID-19 [1]. A large systematic review and meta-analysis of 172,165 pediatric COVID-19 cases found that the presence of underlying conditions greatly increased the odds of critical illness [5]. Specifically, having one comorbidity was associated with about a four-fold higher risk of critical disease, and having multiple comorbidities raised the risk nearly ten-fold. The types of chronic conditions identified as significant risk factors include cardiovascular and neurological disorders, chronic pulmonary diseases (excluding mild asthma), diabetes, obesity, and immunocompromise, all of which were associated with odds ratios above two for severe outcomes. National public health data have echoed these findings: “Children with comorbidities, including asthma, obesity, cystic fibrosis, heart disease, diabetes, and immunosuppression, are at higher risk of severe illness” [2]. Such children have experienced disproportionately severe disease and have been prioritized for vaccination. On the other hand, previously healthy children very rarely develop life-threatening COVID-19 complications, and the absolute risk of critical illness in healthy children is estimated to be around 4% of hospitalized cases [5].

Additional systematic reviews have similarly identified several underlying conditions as important risk factors for severe pediatric COVID-19 [6].

Despite extensive pediatric COVID-19 research, few studies have provided a long-term, population-based description of the burden and severity profile of pediatric COVID-19 hospitalizations across the full course of the pandemic. In particular, nationally representative analyses spanning multiple epidemic phases and several years of follow-up remain scarce [7–10].

This manuscript addresses the following questions. First, what factors predicted moderate-to-severe disease (vs. mild illness) in children hospitalized with COVID-19? We focused on key demographic and clinical predictors such as age and comorbidities. Second, how did the risk profile for moderate-to-severe disease change across different pandemic waves (Pre-Omicron, Omicron BA.1–2, and late Omicron era)? Understanding these patterns can inform clinical risk stratification and guide public health policies to protect the most vulnerable children.

## Methods

### Study design and population

This retrospective observational study used medical data from Maccabi Healthcare Services to examine risk factors for moderate-to-severe disease among hospitalized children aged 0–18 years in Israel. The study covered March 2020 to November 2023, encompassing multiple distinct COVID-19 waves in Israel. The initial dataset included 1,113 pediatric hospitalizations with a confirmed positive SARS-CoV-2 test. Each admission was reviewed to determine whether COVID-19 was the primary reason for hospitalization or an incidental finding. A total of 178 admissions were classified as incidental SARS-CoV-2 infections and were excluded. The final analytic cohort, therefore, included 935 children hospitalized primarily *due* to COVID-19. All included children were younger than 18 years at the time of admission. The research period was divided into three distinct phases: pre-Omicron (February 2020–November 2021), Omicron BA.1–2 (December 2021–May 2022), and late Omicron era (June 2022–November 2023). Figure A and Table A in the Supplementary provide an overview of the COVID-19 waves in Israel, detailing their duration and dominant variants.

The primary estimand of this study was the association between demographic and clinical characteristics and the odds of moderate-to-severe disease among hospitalized children with COVID-19 infection. All regression analyses were therefore conditional on hospitalization and were not intended to estimate risk factors for severe disease in the general infected pediatric population.

### Setting

This retrospective cohort study utilized anonymized data from Maccabi Healthcare Services (MHS), Israel’s second-largest health maintenance organization (HMO), which covers 25% of the population nationwide. In Israel, enrollment in one of four national, state-mandated, non-profit HMOs is compulsory, with citizens free to choose their HMO. By law, HMOs cannot deny membership to any resident. Maccabi’s member base serves as a representative sample of the Israeli population, enabling robust population-based analyses.

### Data collection

Hospitalization records were retrieved from Maccabi Healthcare Services’ centralized electronic databases, which have been maintained for over three decades. The database contains detailed demographic information (age, gender, socioeconomic status, and population group), clinical data (hospitalization severity, comorbidities, outpatient and inpatient diagnoses and procedures), vaccination records, prescribed medications, imaging studies, and comprehensive laboratory results from a single central laboratory.

According to the Israeli Ministry of Health (IMoH), all individuals with a positive SARS-CoV-2 PCR result were mandatorily reported to the national surveillance system, regardless of symptom severity or reason for testing. Hospitals were required to report all hospitalizations of patients testing positive for COVID-19, including their clinical status. The distinction between patients hospitalized *“due to COVID-19”* (i.e., where COVID-19 is the primary cause of admission, typically with respiratory or systemic symptoms) and those hospitalized “with COVID-19” (i.e., incidental SARS-CoV-2 detection during admission for unrelated causes) was made at the hospital level and reported to the IMoH. Medical records of all hospitalizations identified with COVID-19 were manually reviewed to determine whether the admission was attributable to COVID-19. Admissions were classified as COVID-19 related when clinical documentation described respiratory or systemic symptoms consistent with COVID-19. Incidental cases were defined as hospitalizations for unrelated reasons, such as trauma, where COVID-19 was asymptomatic or incidental to the primary diagnosis, and were excluded. The manual review was conducted by an infectious disease specialist.

The primary outcome was disease severity, categorized as either mild or moderate-to-severe during hospitalization. Moderate-to-severe disease was defined as any instance of moderate, severe, critical illness, or death at any point during hospitalization. Severity classification followed the U.S. National Institutes of Health (NIH/CDC) COVID-19 clinical criteria [11] and is detailed in Table B in the Supplementary.

Key covariates included comorbidity (categorized as none, one, two or more), vaccination status (effectively vs. non-vaccinated or ineffectively vaccinated as defined below), age groups (0–1, 1–3, 3–12, 12–15, 15–18), pandemic period (pre-Omicron, Omicron BA.1–2, late Omicron era), socioeconomic status (low, medium, high), and population group (Jewish, Arab, Ultra-Orthodox).

Socioeconomic status (SES) and population sector were classified according to definitions of the Israeli Central Bureau of Statistics [12], based on residential address. SES was categorized into tertiles (low, medium, high) using area-level socioeconomic indices incorporating combined measures of income, education, employment, and housing characteristics, with lower tertiles indicating greater socioeconomic disadvantage.

Population sector (Arab, Ultra-Orthodox Jewish, or general Jewish population) was assigned using address-based classification of residential localities. The Ultra-Orthodox Jewish population represents a distinct religious subgroup in Israel characterized by large household size, high population density, and unique sociodemographic features. These population sectors reflect major sociodemographic groups in Israel that differ in household structure and socioeconomic characteristics.

Vaccination status was defined based on the timing of hospitalization relative to vaccination. Individuals were classified as effectively vaccinated if hospitalized between 14 and 365 days after their second or third vaccine dose, representing the period of active immunity [13].

Comorbidities were based on MHS-validated registries and conditions clinically recognized as predisposing to severe respiratory illness [14–16]. A child was classified as having a comorbidity if they had at least one of the following conditions: diabetes, chronic kidney disease or dialysis dependency, cardiovascular disease (including congenital defects, heart failure), hypertension, or an oncological condition (current or past cancer). Other qualifying conditions include inflammatory bowel disease (Crohn’s or ulcerative colitis), medically complex conditions (such as homebound status, ventilator dependence, or severe disabilities), and chronic respiratory diseases (including asthma). Conditions not considered comorbidities for this study included isolated orthopedic conditions, psychiatric disorders, and elevated body mass index in the absence of other chronic medical conditions. The distribution of specific comorbidities and comorbidity categories is presented in Supplementary Section S7 (Tables S22-23).

### Statistical analysis

To estimate the overall burden of moderate-to-severe disease in the pediatric population in Israel, we used National Insurance reports to calculate rates among all children insured by Maccabi Healthcare Services, not only those who were hospitalized.

All analyses were conducted using R software (version 4.5.0). A fully adjusted multivariable logistic regression model was used to estimate associations between covariates and the odds of moderate-to-severe disease. The model included age group, gender, pandemic period, comorbidities, and vaccination status. All main results reported in the manuscript are derived from this multivariable model. Results are reported as odds ratios (ORs) with corresponding 95% confidence intervals (CIs) and p-values.

In addition to the primary multivariable analysis, we conducted supplementary analyses that included (i) descriptive comparisons of crude data across key subgroups (Supplementary Sections S1–S3), and (ii) sensitivity analyses assessing the robustness of the main findings, including univariable (unadjusted) logistic regression models and a separate analysis of the Delta period (Supplementary Section S5).

Crude Proportions (e.g., rates of moderate-to-severe cases) were estimated with 95% CIs using the Wilson score method.

Multicollinearity among covariates was assessed using variance inflation factors and correlation diagnostics, with particular attention to age group, vaccination status, and pandemic period. In situations of sparse data or complete separation, we fitted penalized logistic regression models using Firth’s correction [17] to reduce small-sample bias and obtain stable odds ratio estimates.

### Ethics statement

This retrospective study was conducted in accordance with the Declaration of Helsinki and approved by the Maccabi Healthcare Services Institutional Review Board (Approval No. MHS-0023-20, approved March 23, 2020).

This study adheres to the Strengthening the Reporting of Observational Studies in Epidemiology (STROBE) guidelines. A completed STROBE checklist is provided in the Supplementary.

## Results

### Study population and descriptive statistics

A total of 1,113 children (aged 0–18 years) were hospitalized with a confirmed SARS-CoV-2 infection during the study period (March 2020 to October 2023). Of these, 178 admissions (16.0%) were classified as incidental SARS-CoV-2 positivity and excluded, leaving a final analytic cohort of 935 children hospitalized due to COVID-19. The distribution of primary versus incidental SARS-CoV-2 admissions by pandemic period is presented in Supplementary Section S8 (Table S24 and Figure S4). Incidental positivity accounted for 18.5% of admissions in the pre-Omicron period, 16.4% during Omicron BA.1–2, and 11.2% in the late Omicron era. Only one admission in the final cohort was identified as multisystem inflammatory syndrome in children (MIS-C). Of the 935 children hospitalized due to COVID-19, 369 admissions occurred in the pre-Omicron period, 320 during Omicron BA.1–2, and 246 in the late Omicron era.

Among the 935 children hospitalized due to COVID-19, 822 (87.9%) were classified as mild. Of the remaining 113 non-mild cases, 57 (6.1% of the full cohort) were classified as moderate, 44 (4.7%) as severe, 11 (1.2%) as critical, and one case (0.1%) resulted in death. Severity distributions stratified by pandemic period are shown in Supplementary Section S9 (Tables S25-26). Overall, 12.1% of hospitalized children experienced moderate-to-severe disease.

Table 1 summarizes the baseline characteristics of the 935 hospitalized children, stratified by disease severity (mild vs. moderate-to-severe). Males were slightly overrepresented across severity groups (54.7% overall). The majority of admissions were among children aged 0–3 years (56.3%), with moderate-to-severe disease most frequently observed in the 1–3-year age group (36.3% of moderate-to-severe cases). Approximately 40% of all admissions had at least one comorbidity, rising to nearly 58% among those with moderate-to-severe disease. Vaccination coverage was low (5.5% overall) and almost absent among moderate-to-severe cases (1.8%). Socioeconomic status was evenly distributed, with 45.1% of children classified as medium SES. Most patients were from the Jewish sector (72.9%), followed by the Ultra-Orthodox Jewish (19.6%) and the Arab (7.5%) sectors. Moderate-to-severe cases were disproportionately concentrated in earlier periods: with 47.8% occurring before the Omicron period, compared to just 17.7% in the late Omicron era.

**Table 1:** Baseline characteristics of the hospitalized study population, stratified by disease severity. The table presents gender, age group, period, socioeconomic status (SES), population group distribution, presence of comorbidities, and vaccination status. Data are reported as counts and percentages

		Mild	Moderate-to-Severe	Overall
Gender	Females	382 (46.5%)	42 (37.2%)	424 (45.3%)
	Male	440 (53.5%)	71 (62.8%)	511 (54.7%)
Age group	0-1	229 (27.9%)	11 (9.7%)	240 (25.7%)
	1-3	245 (29.8%)	41 (36.3%)	286 (30.6%)
	3-12	198 (24.1%)	27 (23.9%)	225 (24.1%)
	12-15	59 (7.2%)	15 (13.3%)	74 (7.9%)
	15-18	91 (11.1%)	19 (16.8%)	110 (11.8%)
Comorbidity	0	503 (61.2%)	48 (42.5%)	551 (58.9%)
	1	295 (35.9%)	51 (45.1%)	346 (37.0%)
	2+	24 (2.9%)	14 (12.4%)	38 (4.1%)
Vaccination	Unvaccinated	773 (94.0%)	111 (98.2%)	884 (94.5%)
	Vaccinated	49 (6.0%)	2 (1.8%)	51 (5.5%)
SES	Low	145 (17.6%)	24 (21.2%)	169 (18.1%)
	Medium	375 (45.6%)	47 (41.6%)	422 (45.1%)
	High	302 (36.7%)	42 (37.2%)	344 (36.8%)
Population Group	Jewish	608 (74.0%)	74 (65.5%)	682 (72.9%)
	Arab	58 (7.1%)	12 (10.6%)	70 (7.5%)
	UltraOrthodox	156 (19.0%)	27 (23.9%)	183 (19.6%)
Period	Pre-Omicron	315 (38.3%)	54 (47.8%)	369 (39.5%)
	Omicron BA.1–2	281 (34.2%)	39 (34.5%)	320 (34.2%)
	Late Omicron era	226 (27.5%)	20 (17.7%)	246 (26.3%)
Total		822 (87.9%)	113 (12.1%)	935 (100%)

#### Population-level hospitalization rates

Among 769,607 children covered by Maccabi, 935 were hospitalized for COVID-19, including 113 classified as moderate-to-severe cases. This corresponds to an overall hospitalization rate of 33.9 per 100,000 children per year (95% CI: 31.8–36.1) and a moderate-to-severe disease rate of 4.1 per 100,000 children per year (95% CI: 3.4–4.9). Additional details, including age-stratified rates of hospitalization and moderate-to-severe disease, are provided in Section S4 of the Supplementary.

### Risk factors for moderate-to-severe illness

**Fig. 1:**
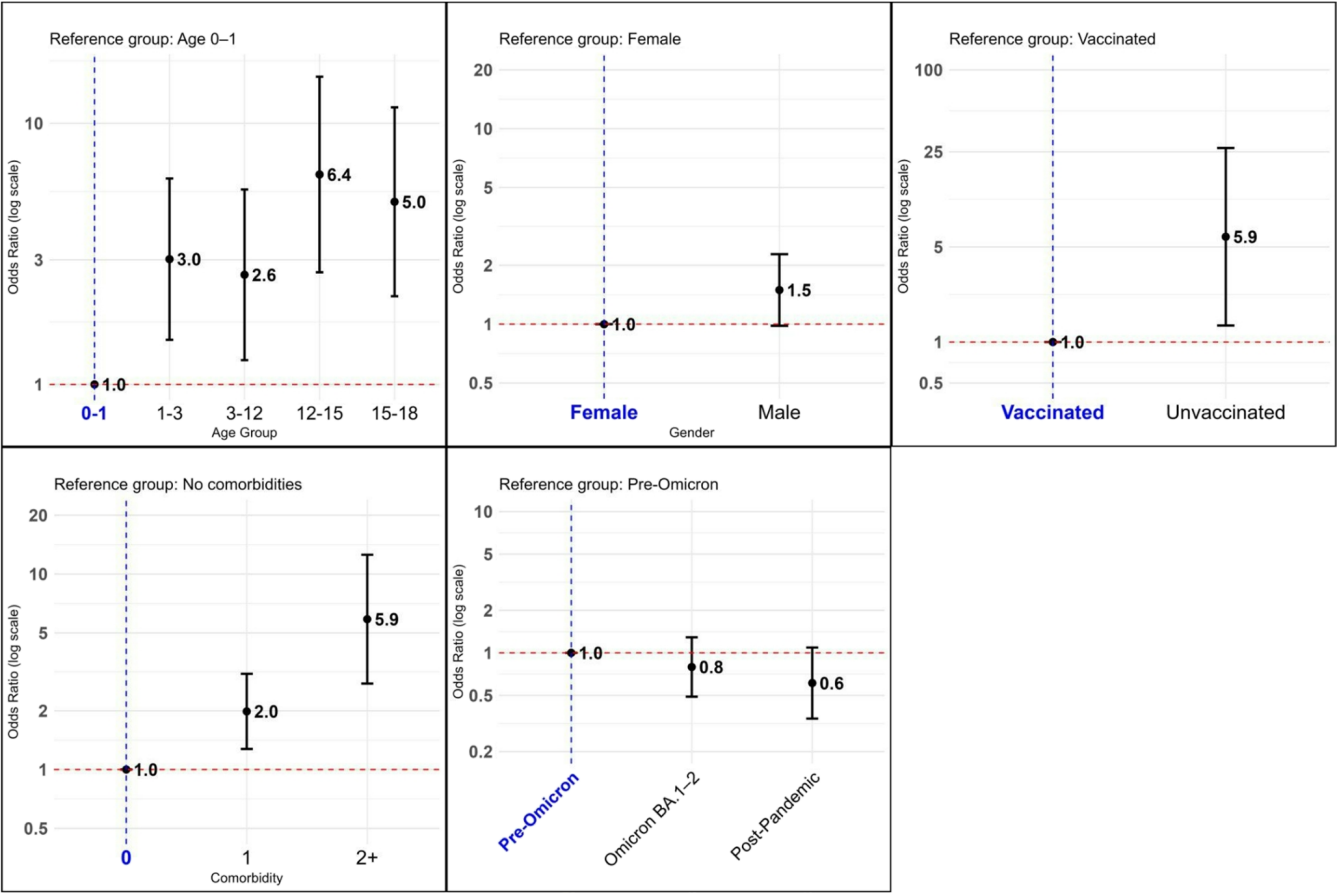
The main risk factors found in the analysis. Adjusted odds ratios (ORs) and 95% confidence intervals for moderate-to-severe disease among hospitalized children, obtained from the multivariable logistic regression model covering the study period from May 1, 2020, to November 30, 2023. Estimates are adjusted for age group, gender, pandemic period, comorbidity, and vaccination status. Confidence intervals are not adjusted for multiplicity. The reference level of each covariate is shown in blue. Note that the y-axis is on a logarithmic scale

A fully adjusted multivariable logistic regression model was fitted to identify factors associated with moderate-to-severe COVID-19 among hospitalized children. The model simultaneously adjusted for age group, sex, pandemic period, comorbidity burden, and vaccination status. Adjusted odds ratios from this model are presented in Table 2; Fig. 1.

**Table 2:** Multivariable-adjusted logistic regression model for moderate-to-severe disease among hospitalized children (*n* = 935), presenting adjusted odds ratios by age group, gender, pandemic period, comorbidity, and vaccination status

Predictors	Category	Odds Ratios	CI	*p*-value
Gender	Female	1	*Reference Level*	
	Male	1.5	0.98–2.28	0.061
Period	Pre-Omicron	1	*Reference Level*	
	Omicron BA.1–2	0.79	0.49–1.29	0.351
	Late Omicron era	0.61	0.34–1.09	0.096
Comorbidity	0	1	*Reference Level*	
	1	1.98	1.27–3.09	0.002
	“+2”	5.88	2.75–12.55	< 0.001
Age group	0–1	1	*Reference Level*	
	1–3	3.02	1.48–6.14	0.002
	3–12	2.63	1.24–5.58	0.012
	12–15	6.36	2.69–15.07	< 0.001
	15–18	5	2.18–11.50	< 0.001
Vaccination status	Vaccinated	1	*Reference Level*	
	Unvaccinated	5.94	1.32–26.66	0.02

Multicollinearity among covariates was assessed using variance inflation factors and correlation diagnostics, with particular attention to age group, vaccination status, and pandemic period. No evidence of problematic multicollinearity was observed (all adjusted GVIF values < 1.05). Correlation diagnostics between key categorical variables indicated only weak to moderate associations (Cramer’s V: age × period = 0.22; age × vaccination = 0.38; period × vaccination = 0.16), with no indication of numerical instability. The results below describe the associations for each covariate included in the model.

#### Comorbidity burden

Comorbidity burden was the strongest predictor of moderate-to-severe disease. Compared with children without comorbidities, those with one comorbidity had nearly twice the odds of moderate-to-severe illness (OR = 1.98; 95% CI: 1.27–3.09; *p* = 0.002), while those with two or more comorbidities had nearly sixfold higher odds (OR = 5.88; 95% CI: 2.75–12.55; Table 2).

#### Vaccination status

Unvaccinated children were associated with higher odds of moderate-to-severe disease (OR = 5.94; 95% CI: 1.32–26.66). Because vaccination was rare, penalized logistic regression using Firth’s correction [17] was applied, yielding a similar magnitude and direction of association (OR = 4.77; 95% CI: 1.25–18.17; Supplementary Table S14).

To address age-related vaccine eligibility, analyses were repeated among vaccine-eligible children aged 5–18 years (*n* = 338). In this subgroup, lack of vaccination remained associated with higher odds of moderate-to-severe disease (OR = 5.22; 95% CI: 1.13–24.19), with consistent estimates in the penalized model (OR = 4.16; 95% CI: 1.09–15.96; Supplementary Tables S9–S10). Event counts, absolute risks, and absolute risk differences by vaccination status are provided in Supplementary Tables S18 and S19.

#### Age group

Age was associated with disease severity. Relative to infants aged 0–1 years, children aged 1–3 years had approximately threefold higher odds of moderate-to-severe disease (OR = 3.02; 95% CI: 1.48–6.14), while children aged 3–12 years had 2.6-fold higher odds (OR = 2.63; 95% CI: 1.24–5.58). The highest odds were observed among adolescents aged 12–15 years (OR = 6.36; 95% CI: 2.69–15.07) and 15–18 years (OR = 5.00; 95% CI: 2.18–11.50). At the population level, infants had the highest hospitalization rates, whereas children aged 1–3 years had the highest rates of moderate-to-severe disease (Supplementary Table S5).

#### Gender

Males were associated with increased odds of moderate-to-severe disease compared to females, though this did not reach statistical significance (OR = 1.50; 95% CI: 0.98–2.28; *p* = 0.061).

#### Pandemic period

Compared with the pre-Omicron period, the odds of moderate-to-severe disease among hospitalized children were lower during the Omicron BA.1–2 wave and the late Omicron era, although these differences did not reach statistical significance (OR = 0.79, 95% CI: 0.49–1.29, *p* = 0.351; OR = 0.61, 95% CI: 0.34–1.09, *p* = 0.096).

#### Additional crude data analyses

In addition to the regression-based analyses described above, descriptive analyses based on crude data comparing disease severity across key subgroups are presented in Supplementary Sections S1–S3, providing complementary context to the adjusted associations.

#### Stricter outcome definition

The main analysis was repeated using a stricter outcome definition, defining “severe” as severe/critical disease or death (versus mild/moderate). In this analysis, the associations with comorbidity and older age remained strong. Regarding vaccination, no severe events occurred among vaccinated children, resulting in complete separation in standard logistic regression and making estimation of odds ratios for vaccination impossible (Supplementary Table S20). In penalized logistic regression using Firth’s correction [17], lack of vaccination was associated with substantially higher odds of severe disease (OR = 15.15; Table S21), although confidence intervals were wide, reflecting limited statistical power. Additional details are provided in Supplementary Section S6 (Tables S16-S21).

#### Additional sensitivity analyses

Additional sensitivity analyses examining the robustness of the main findings are presented in Supplementary Section S5, including analyses adjusting for population group and socioeconomic status, subgroup analyses, alternative variable definitions, and univariable (unadjusted) logistic regression models (Table S13). These also include a sensitivity analysis separating the Delta wave from earlier pre-Omicron months (Table S15).

## Discussion

This retrospective cohort study analyzed all COVID-19 hospitalizations among children aged 0–18 years from March 2020 to November 2023 in Maccabi Healthcare Services, which provides care to approximately 770,000 children, nearly a quarter of Israel’s pediatric population. The relatively low number of hospitalizations (935 cases) in this large and representative population underscores the rarity of moderate-to-severe COVID-19 requiring hospitalization in children. Our analysis, spanning the pre-Omicron, Omicron BA.1–2, and late Omicron eras, identified key factors associated with moderate-to-severe pediatric COVID-19. Overall, moderate-to-severe disease was uncommon, accounting for 12.1% of hospitalizations, with annualized rates of 33.9 hospitalizations and 4.1 moderate-to-severe cases per 100,000 children. Given the retrospective observational design, all findings should be interpreted as associations rather than causal effects.

Comorbidities were the strongest predictor of disease severity; over 50% of severe cases had at least one underlying condition, and the presence of multiple comorbidities was associated with nearly sixfold higher odds of severe disease. While previous meta-analyses emphasize a broad range of underlying conditions [18], our data show that respiratory diseases and asthma represent the vast majority of these comorbidities. This underscores the importance of monitoring children with common respiratory vulnerabilities, as they constitute a significant portion of the population at risk for severe outcomes.

Among hospitalized infants (aged 0–1 years), 10 of 128 males and 1 of 112 females experienced moderate-to-severe disease. Although this difference is unlikely to be explained by chance alone, these findings are based on very small event counts, and the resulting estimates are highly imprecise and should not be overinterpreted (Supplementary Table S8).

Age-related patterns were more nuanced. Although older hospitalized children, particularly adolescents, had higher odds of moderate-to-severe disease within the hospitalized cohort, this did not translate into a higher population-level burden. Instead, the absolute burden of both hospitalization and severe disease was concentrated in early childhood. Infants had the highest hospitalization rates, while children aged 1–3 years showed the highest population-level rates of severe outcomes. Importantly, hospitalization decisions in infants and very young children are often influenced by precautionary clinical thresholds and the need for observation rather than disease severity alone. This practice may lead to overrepresentation of milder illness among hospitalized infants and introduces age-related admission bias. This highlights the importance of distinguishing between relative odds within hospitalized patients and absolute population-level rates when interpreting age-related patterns of severe pediatric COVID-19. Moreover, assessing trends in severity over time is inherently challenging due to concurrent changes in viral variants, population immunity, and clinical and public health practices.

During the study period, we observed a descriptive decline in the proportion of moderate-to-severe cases among hospitalized children from the pre-Omicron to the late Omicron era. While these temporal trends suggest a shift toward lower clinical severity over time, the adjusted period effects remain statistically uncertain, and the observed patterns likely reflect a complex interplay of several factors. These include increasing population immunity, derived from both vaccination and cumulative prior infections, as well as potential shifts in variant-specific characteristics and evolving clinical admission thresholds.

International data have similarly noted declining pediatric severity across successive waves, reinforcing the view that multiple concurrent influences shape these trends [19–21]. Furthermore, changes in testing practices and hospital management strategies, particularly following the emergence of Omicron, may have contributed to a perceived reduction in severity among the hospitalized cohort [22].

While some evidence suggests that Omicron BA.1–2 resulted in fewer critical outcomes compared to the Delta variant in pediatric populations [23, 24], our findings should be interpreted as a descriptive reflection of the shifting risk landscape rather than a definitive indication of reduced intrinsic viral virulence. Because each study period encompasses simultaneous changes in variant predominance, rising immunity, and shifting healthcare practices, these trends cannot be causally attributed to any single factor. The specific limitations of assessing longitudinal shifts in severity are detailed in the limitations section below.

Vaccination was associated with lower odds of moderate-to-severe disease. However, only 51 hospitalized children in the cohort were vaccinated, resulting in wide confidence intervals and limited statistical precision (OR 5.94, 95% CI 1.32–26.66). While vaccination was associated with lower odds of severe disease, the magnitude of this association remains uncertain. Moreover, measuring such effects in a retrospective observational analysis is inherently prone to bias (e.g., confounding by indication and healthy vaccinee bias); these findings should be interpreted cautiously. Accordingly, we conducted two sensitivity analyses (analyses 5 and 6 in Supplementary Section S5) to test the robustness of our findings under alternative definitions of vaccination status. In the first, using a narrower window (14 days to 6 months post-dose), the association with reduced odds of moderate-to-severe disease was weaker and no longer statistically significant, likely due to reduced sample size rather than a true decline in effectiveness. Notably, associations for other predictors, including comorbidity and age, remained stable (Supplementary Table S11). In the second, using a broader definition that included children who received only a single dose, the association remained statistically significant, though the estimated effect was smaller, potentially reflecting the more limited and variable protection conferred by a single dose [25]. Associations for comorbidity, age group, and pandemic period remained consistent (Supplementary Table S12). Together, these analyses highlight both the consistency in the direction of the association and the uncertainty in its estimation. Interestingly, when further restricting the outcome to severe, critical illness, or death, the main associations with comorbidity and age remained consistent. However, the absence of severe outcomes among vaccinated children resulted in complete separation and wide confidence intervals, highlighting the limited precision for estimating vaccine-associated effects under stricter outcome definitions (Supplementary Tables S20-S21).

Our findings are broadly consistent with reports from Israel and European settings showing that pediatric COVID-19 hospitalizations and severe outcomes were uncommon, while underlying medical conditions were consistently associated with higher severity among hospitalized children. In Israel, national hospital-based surveillance early in the pandemic similarly documented that most pediatric cases were mild, with severe outcomes concentrated in a minority of hospitalized children [26]. In European cohorts, pediatric COVID-19 hospitalizations were likewise rare, with comorbidities consistently overrepresented among more severe outcomes [8]. Studies from Israel and several European countries have reported higher hospitalization rates among the youngest children, frequently attributed in part to precautionary admission thresholds in infants rather than disease severity alone [10]. These findings highlight that age-related patterns derived from hospitalized cohorts are strongly influenced by admission practices and should be interpreted primarily in the context of population-level hospitalization and severe disease rates.

While this study provides insight into risk factors for moderate-to-severe disease among hospitalized children, it has several limitations. The retrospective observational design is inherently prone to bias, including possible misclassification of severity, small sample sizes in subgroups, and residual confounding. In particular, the vaccination analysis was based on a small number of vaccinated hospitalized children (*n* = 51), resulting in wide confidence intervals and limited statistical power to estimate the magnitude of vaccine-associated protection precisely. Residual confounding, including healthy vaccinee bias and unmeasured differences between vaccinated and unvaccinated children, cannot be excluded. An important unmeasured confounder is prior SARS-CoV-2 infection, for which no individual-level data were available in our dataset. By the late Omicron era, a large proportion of children are likely to have been previously infected, resulting in substantial infection-induced or hybrid immunity [27]. This is expected to bias period comparisons toward lower observed severity in later periods, independent of intrinsic differences in variant virulence. Consequently, declines in severity over time should not be attributed to viral characteristics alone. In addition, apparent associations with vaccination status may partly reflect differences in prior infection history, with unvaccinated children more likely to have acquired immunity through previous SARS-CoV-2 infection.

Although we distinguished between hospitalizations due to COVID-19 and incidental SARS-CoV-2–positive admissions, we did not have detailed severity classification data for incidental cases and therefore could not compare severity distributions between primary and incidental hospitalizations. Accordingly, all severity analyses in this study were restricted a priori to children hospitalized due to COVID-19.

Furthermore, the regression analyses of risk factors were conditional on hospitalization. Conditioning on hospitalization may induce collider-selection bias, because factors such as comorbidity burden, young age, vaccination status, and pandemic period influence both the probability of being hospitalized and the probability of experiencing more severe disease. Consequently, associations observed within the hospitalized cohort may be distorted in magnitude or even direction. For example, children with chronic conditions may be admitted with milder illness due to lower admission thresholds, potentially biasing observed associations between comorbidity and severity. Therefore, the reported regression associations should not be assumed to reflect the underlying relationships even within the hospitalized cohort, nor extrapolated beyond hospitalized children. However, this limitation applies specifically to the regression analyses of severity among hospitalized children and does not pertain to the population-level analysis, which was estimated using denominators from the full Maccabi pediatric population (see Section S4 of the Supplementary).

Finally, this study was conducted within a single large healthcare organization in Israel and included only hospitalized children. Differences in healthcare systems, testing policies, admission thresholds, population structure, and background immunity may limit the generalizability of these findings to other settings or pediatric populations.

Our findings suggest three key points: first, COVID-19 in children was predominantly mild at the population level across successive pandemic periods, with a minimal burden of severe disease. Second, despite this overall pattern, the absolute number of pediatric COVID-19 hospitalizations was not negligible and continues to warrant clinical and policy attention. Third, in the current endemic phase, characterized by widespread background immunity and lower hospitalization rates than earlier in the pandemic, the consistently strong association between comorbidity burden and disease severity remains evident. This association supports a risk-stratified approach, in which preventive efforts and early intervention are prioritized for children with chronic medical conditions rather than applied uniformly across the pediatric population. Conversely, the low absolute rates of hospitalization and moderate-to-severe disease among otherwise healthy children suggest that broad, non-targeted interventions are likely to yield limited additional benefit in high-immunity settings.

## Supplementary Information

Below is the link to the electronic supplementary material.

Supplementary Material 1

## Data Availability

The data supporting this study are available from the corresponding author, but restrictions apply to the availability of such information. It was used under a license for the current study and is not publicly available. Data are, however, available from the authors upon reasonable request and with permission of the local ethics committee of MHS.

## Abbreviations

COVID-19: Coronavirus Disease 2019
MIS-C: Multisystem Inflammatory Syndrome in Children
MHS: Maccabi Healthcare Services
HMO: Health Maintenance Organization
SES: Socioeconomic Status
NPI: Non-Pharmaceutical Intervention
OR: Odds Ratio
WHO: World Health Organization
