# Supplementary material for: Disease severity among hospitalized children during the COVID-19 pandemic in Israel
Source: Eur J Clin Microbiol Infect Dis. 2026 Apr 9;45(8):2225–34. doi: 10.1007/s10096-026-05485-6 (PMC13428773; doi:10.1007/s10096-026-05485-6)
Supplement: Supplementary file 1 — Supplementary Material 1 [file 10096_2026_5485_MOESM1_ESM.pdf]

Disease severity among hospitalized children during the COVID-19 pandemic in Israel

Supplementary Information

Ilan Livne, Ph.D., The Gertner Institute for Epidemiology & Health Policy Research, Sheba Medical Center; Samuel Neaman Institute for National Policy Research, Technion – Israel Institute of Technology, Israel.

Yair Goldberg[&], Ph.D., Technion - Israel Institute of Technology.

Amit Huppert[&], Ph.D., Data & Analytics, Sheba Medical Center and Tel Aviv University.

Michal Stein[&], MD, Pediatric Infectious Disease Unit, The Edmond and Lily Safra Children's Hospital, Chaim Sheba Medical Center, Ramat Gan, Israel; Faculty of Medical and Health Sciences, Tel Aviv University, Tel Aviv, Israel.

Shirley Shapiro Ben David[&], MD, Maccabi Healthcare Systems, Division of Health, Infectious Disease Unit, Tel Aviv, Israel; Faculty of Medical and Health Sciences, Tel Aviv University, Tel Aviv, Israel.

[&] Contributed equally

Corresponding author:
Ilan Livne, Samuel Neaman Institute for National Policy Research, Technion City, Haifa 3200003, Israel
Phone: +972-73-3785870
Email: ilan@sni.technion.ac.il

Table A: The period, the wave, the start date, the end date, the number of days, and the dominant variant of each wave in the study period.

Period	Wave Number	Start Date	End Date	Number of Days	Dominant COVID-19 Variants
Per-Omicron	Wave 1	1/3/2020	30/5/2020	90	Wuhan
	Wave 2	1/6/2020	30/11/2020	182	Wuhan
	Wave 3	1/12/2020	14/6/2021	195	Alpha
	Wave 4	15/6/2021	14/12/2021	182	Delta
Omicron	Wave 5	15/12/2021	14/5/2022	150	Omicron BA.1-2
Late Omicron era	Wave 6	15/5/2022	30/9/2022	138	Omicron BA.5
	Wave 7	1/10/2022	14/2/2023	136	Omicron BQ.1
	Wave 8	15/2/2023	14/6/2023	119	Omicron Xbb.1.5
	Wave 9	15/6/2023	30/11/2023	168	Omicron Xbb1.9 & EG.5.1

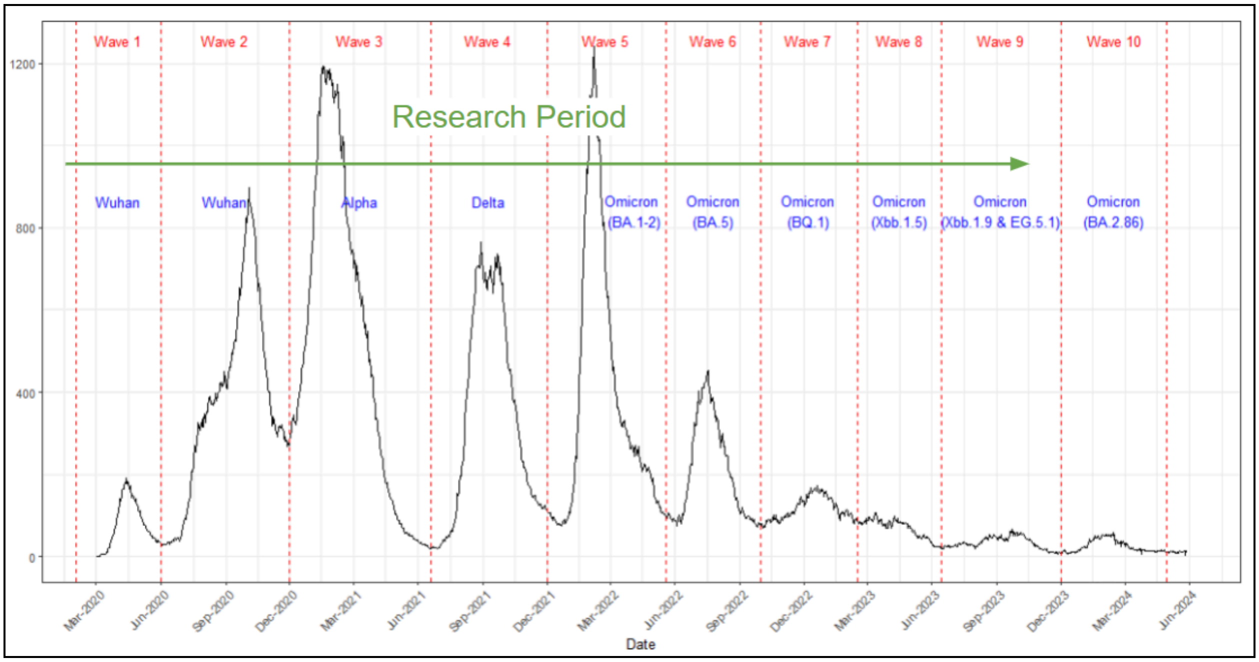

Figure A. *The number of severe COVID-19 cases in Israel over time. The research period of this study, from March 2020 to November 2023, is indicated by a green arrow. Dominant variants for each wave are shown in blue.*

Table B: Classification of COVID-19 severity (mild, moderate, severe, critical) based on NIH/CDC criteria, adapted for pediatric patients, including clinical features and respiratory parameters. Criteria derived from CDC interim guidance for SARS-CoV-2 infection (2022), with pediatric modifications accounting for age-specific respiratory physiology.

Severity Level	Clinical Features & Symptoms	Oxygen Saturation (SpO2) & Respiratory Metrics
**Mild Illness**	Fever, cough, sore throat, malaise, headache, muscle pain. **No** shortness of breath or abnormal imaging.	Normal / Not specified
**Moderate Illness**	Evidence of lower respiratory disease (via clinical assessment or imaging).	≥ 94% on room air (at sea level)
**Severe Illness**	Lung infiltrates >50%. In children, **hypoxia is the primary criterion**, as normal respiratory rates vary by age.	SpO_2 < 94% (or >3% drop from baseline); Resp rate >30 bpm; PaO_2/FiO_2 < 300 mmHg
**Critical Illness**	Respiratory failure, septic shock, and/or multiple organ dysfunction.	Requires mechanical ventilation or life support

Supplementary S1–S3: Descriptive Analyses Based on Crude Data

S1: Analysis by Periods (Crude Data)

Among the 935 hospitalized children, 113 (113/935 = 12.1%, 95% CI: 10.1%–14.3%) experienced moderate-to-severe disease over a total of 1,315 days. The pre-Omicron period showed the highest proportion of moderate-to-severe cases (54/369, 14.6%, 95% CI: 11.4%–18.6%), followed by the Omicron BA.1-2 (39/320, 12.2%, 95% CI: 9.0%–16.2%) and late Omicron era (20/246, 8.1%, 95% CI: 5.3%–12.2%) periods. However, the overlapping confidence intervals suggest that these differences are not statistically significant. To account for differences in period length, we calculated the average number of hospitalizations and moderate-to-severe cases per month (Table S2). The Omicron BA.1-2 period had the highest averages, with 64.0 hospitalizations and 7.8 moderate-to-severe cases per month, compared to 17.0 and 2.5 pre-Omicron, and 14.3 and 1.2 late Omicron era, respectively. Overall, the average monthly hospitalization count was 21.3, and the average number of moderate-to-severe cases was 2.6 (Table S1).

Figure S1 illustrates these trends, showing a sharp increase in hospitalizations during the Omicron period, with both mild and moderate-to-severe cases concentrated in the early phase of the wave. moderate-to-severe cases, though less frequent overall, were observed across all periods, with the highest concentration during the early Omicron BA.1-2 period. In the post-Omicron period, hospitalizations declined substantially, and moderate-to-severe cases became rare. A corresponding figure stratified by age group is provided in Figure S2.

Table S1*. Hospitalization and moderate-to-severe case rates across different pandemic periods. The table presents the number of hospitalized patients, moderate-to-severe cases, and corresponding moderate-to-severe case rates (with 95% confidence intervals) during the pre-Omicron, Omicron BA.1-2, and late Omicron periods. Additionally, daily hospitalization and moderate-to-severe case rates per period are reported.*

Category	Pre-Omicron	Omicron BA.1-2	Late Omicron era	Total
Number of Hospitalized	369	320	246	**935**
Number of Moderate-to-Severe	54	39	20	**113**
Moderate-to-Severe Case Rate (95% CI)	14.6% (CI: 11.4%, 18.6%)	12.2% (CI: 9%, 16.2%)	8.1% (CI: 5.3%, 12.2%)	**12.1% (CI: 10.1%, 14.3%)**
Period Length (months)	21.7	5	17.2	**43.9**
Average Number of Hospitalizations per Month	17.0	64.0	14.3	**21.3**
Average Number of Moderate-to-Severe Cases per Month	2.5	7.8	1.2	**2.6**

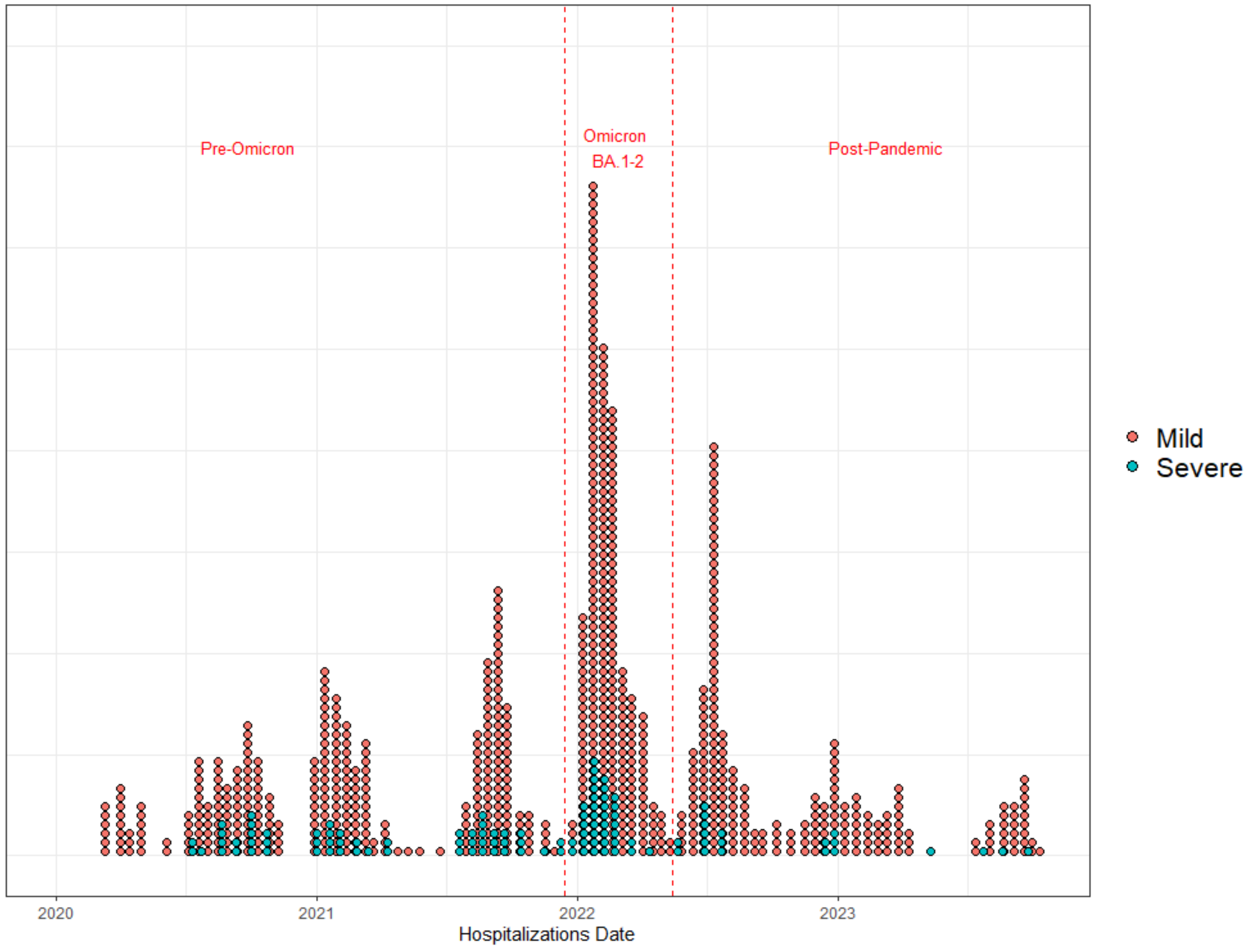

Figure S1*: Hospitalizations over time by severity. The X-axis represents hospitalization dates, grouped into 14-day bins. Each dot represents a single hospitalization, and dots are stacked vertically when multiple hospitalizations occurred within the same 14-day window.*

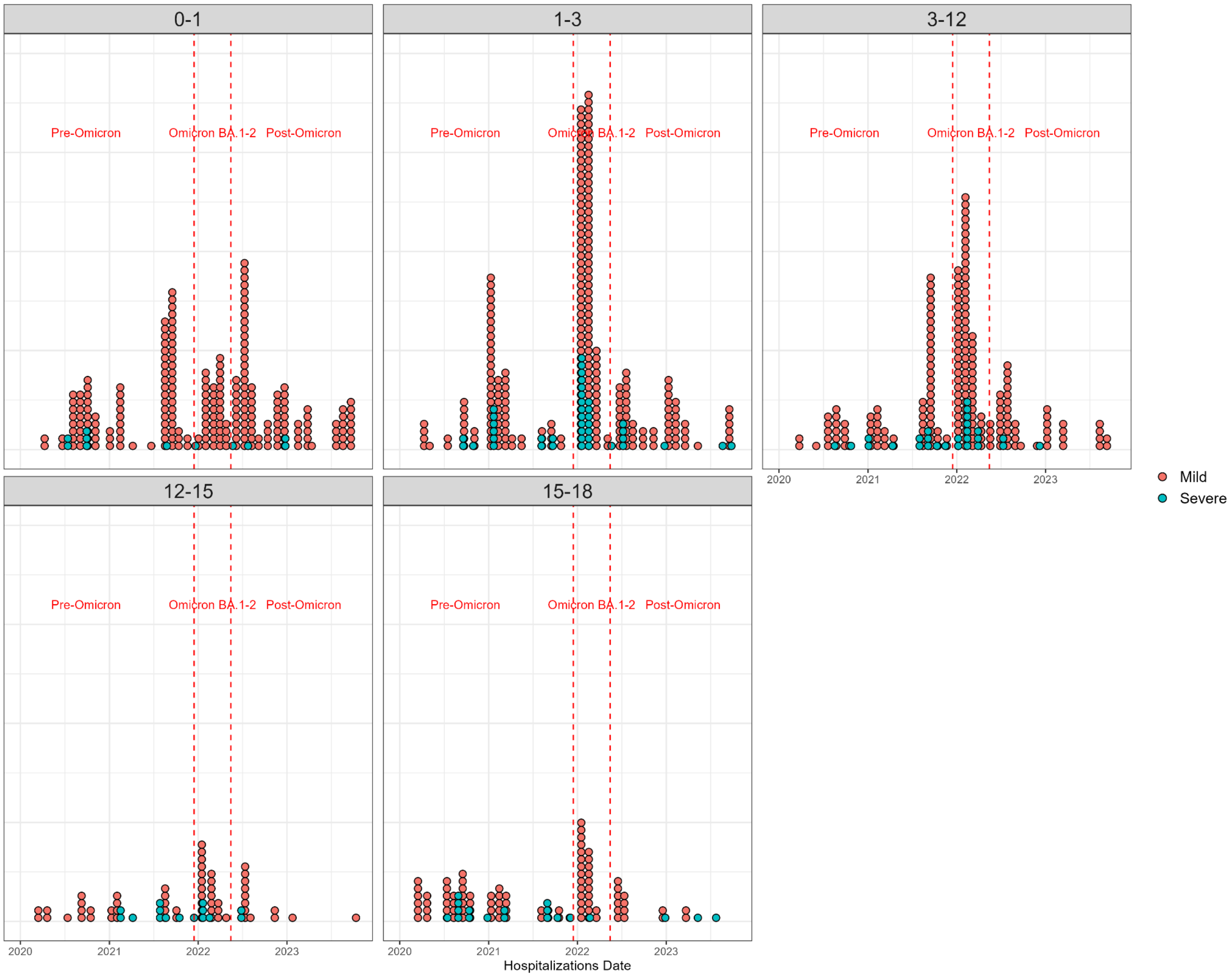

Figure S2. *Distribution of hospitalization cases over time by age group. The figure presents the timing of mild (red) and moderate-to-severe (blue) hospitalizations across four age groups (0-1,1–3, 3–12, 12–15, and 15–18 years). Vertical dashed red lines indicate the start and end of the Omicron BA.1-2 wave, dividing the pre-Omicron, Omicron BA.1-2, and late Omicron periods.*

S2: Analysis by Age Group and Comorbidity Status (Crude Data)

We analyzed disease severity across age groups (0-1, 1–3, 3–12, 12–15, and 15–18 years) among 935 hospitalized children (Table S2). The highest proportion of moderate-to-severe cases was observed in the 12–15 age group (20.3%, 95% CI: 12.7%–30.8%), followed by 15–18 years (17.3%, 95% CI: 11.3%–25.4%), while infants had the lowest rate (4.6%, 95% CI: 2.6%–8.0%). Although older children appeared to have higher moderate-to-severe disease rates, overlapping confidence intervals suggest these differences may not be statistically significant.

In addition to age, we examined the impact of comorbidities on disease severity. moderate-to-severe illness was more common in children with comorbidities (16.9%, 95% CI: 13.5%–21.0%) compared to those without (8.7%, 95% CI: 6.6%–11.4%), nearly doubling the risk. A Chi-square test confirmed a statistically significant association between comorbidities and disease severity ($\chi^2 = 13.61$, $p = 0.0002$).

Further stratification by age revealed a consistent pattern of higher moderate-to-severe disease rates among children with comorbidities across all age groups. While this increased risk was apparent, overlapping confidence intervals, likely due to small subgroup sizes, suggest that not all differences may be statistically significant. Nonetheless, the overall trend reinforces the heightened vulnerability of children with comorbidities, particularly in older age groups (see Table S3 and Figure S3).

Table S2. *Distribution of moderate-to-severe cases among hospitalized children by age group. The table presents the number of hospitalized children, the number of moderate-to-severe cases, and the proportion of moderate-to-severe cases with 95% confidence intervals (CI).*

Age Group	Number of Hospitalized	Number of moderate-to-severe Cases	moderate-to-severe Case Rate (95% CI)
0-1	240	11	4.6% (95% CI: 2.6%, 8%)
1-3	286	41	14.3% (95% CI: 10.7%, 18.9%)
3-12	225	27	12% (95% CI: 8.4%, 16.9%)
12-15	74	15	20.3% (95% CI: 12.7%, 30.8%)
15-18	110	19	17.3% (95% CI: 11.3%, 25.4%)
**Total**	**935**	**113**	**12.1% (95% CI: 10.1%, 14.3%)**

Table S3. *Moderate-to-Severe Disease Risk Among Hospitalized Children with Underlying Conditions*
This table shows the number of hospitalized children with underlying conditions and the proportion who developed moderate-to-severe disease, stratified by age group.

	Comorbid			Not comorbid		
**Age Group**	**Hospitalized**	**Moderate-to-Severe Cases**	**Moderate-to-Severe Case Rate (95% CI)**	**Hospitalized**	**Moderate-to-Severe Cases**	**Moderate-to-Severe Case Rate**
0-1	94	7	7.4% (95% CI: 3.7%, 14.6%)	146	4	2.7% (95% CI: 1.1%, 6.8%)
1-3	149	25	16.8% (95% CI: 11.6%, 23.6%)	137	16	11.7% (95% CI: 7.3%, 18.1%)
3-12	85	17	20% (95% CI: 12.9%, 29.7%)	140	10	7.1% (95% CI: 3.9%, 12.6%)
12-15	24	7	29.2% (95% CI: 14.9%, 49.2%)	50	8	16% (95% CI: 8.3%, 28.5%)
15-18	32	9	28.1% (95% CI: 15.6%, 45.4%)	78	10	12.8% (95% CI: 7.1%, 22%)
**Total**	**384**	**65**	**16.9% (95% CI: 13.5%, 21%)**	**551**	**48**	**8.7% (95% CI: 6.6%, 11.4%)**

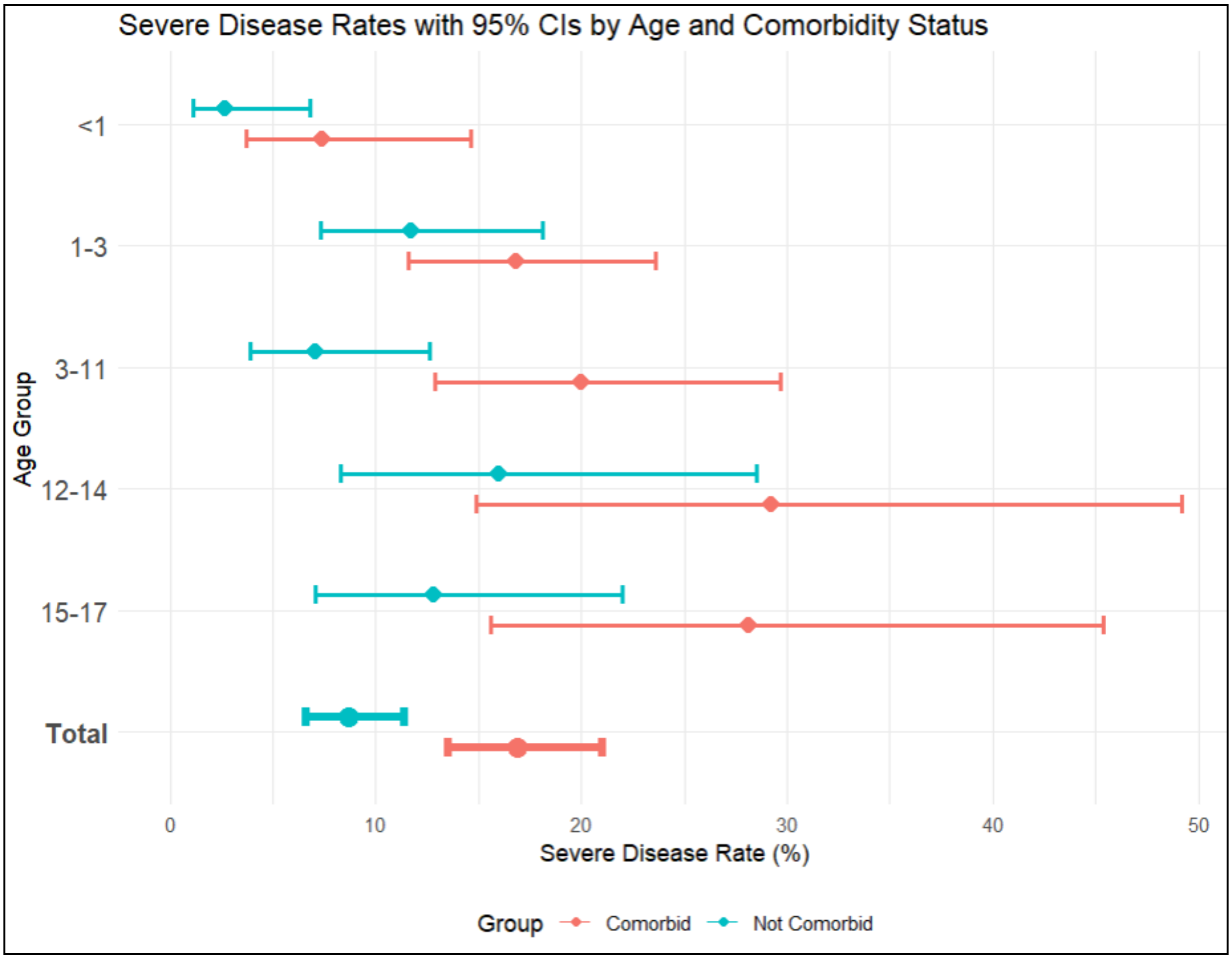

Figure S3: *Moderate-to-severe COVID-19 disease rates among hospitalized children by age group and comorbidity status, with 95% confidence intervals.*

S3: Analysis by Vaccination Status (Crude Data)

We examined the association between vaccination status and disease severity among hospitalized children, restricting the analysis to those aged 6 years and older (n=338), as vaccination coverage among younger children (0–5 years) was negligible in practice. Moderate-to-severe illness was significantly less common in vaccinated children (3.9%, 95% CI: 1.08%–13.2%) compared to unvaccinated children (17.1%, 95% CI: 13.2%–21.9%), reflecting a fourfold difference in risk. A Chi-square test ($\chi^2 = 5.55$, $p = 0.018$) indicated a statistically significant association between vaccination and reduced disease severity. A consistent pattern of lower moderate-to-severe disease rates was observed among vaccinated children across all age groups, periods, and comorbidity status (Table S4).

Table S4*. Moderate-to-severe COVID-19 cases among hospitalized children aged 5–18, stratified by vaccination status and (a) age group, (b) pandemic period, and (c) comorbidity status. The table presents the number of hospitalizations, moderate-to-severe cases, and corresponding moderate-to-severe case rates, with 95% confidence intervals shown for the total estimates in each subgroup.*

	Unvaccinated			Vaccinated		
**Group**	Hospitalized	Moderate-to-severe Cases	Moderate-to-severe Case Rate	Hospitalized	Moderate-to-severe Cases	Moderate-to-severe Case Rate
0-5	**Vaccination Coverage negligible; excluded from comparison**					
5-12	144	16	11.1%	10	1	10.0%
12-15	60	14	23.3%	14	1	7.1%
15-18	83	19	22.9%	27	0	0%
Pre-Omicron	157	33	19.8%	4	0	0.0%
Omicron BA.1-2	86	9	13.4%	29	2	6.9%
Late Omicron era	44	7	12.9%	18	0	0.0%
Non-Comorbid	199	25	12.0%	34	0	0.0%
Comorbid	88	24	25.0%	17	2	11.8%
**Total**	**287**	**49**	**17.1% (95% CI: 13.2%–21.9%)**	**51**	**2**	**3.9% (95% CI: 1.08%–13.2%)**

Supplementary S4: Estimating the Hospitalization Rate and Moderate-to-Severe Case Rate in the Population Using Additional Information from National Insurance Reports

In this section, we estimate the overall burden of moderate-to-severe disease in the *entire population* by including all children covered by Maccabi Healthcare Services, not just those who were hospitalized. Data on the total child population in Maccabi were obtained from National Insurance reports for the years 2020 and 2023 [1,2]. The number of children at each age was calculated as the average of the figures reported for these two years.

Table S5 presents the total number of children, hospitalizations, and moderate-to-severe cases, along with their corresponding rates per 100,000 children per year across five age groups (<1, 1–3, 3–12, 12–15, and 15–18). The data indicate that younger children, particularly those under age 1, have the highest hospitalization rates (156.4 per 100,000 per year, 95% CI: 137.8–177.4) compared to older age groups. However, the highest moderate-to-severe case rate is observed in the 1–3 age group (12.9 per 100,000 per year, 95% CI: 9.5–17.6), while other age groups exhibit lower moderate-to-severe disease rates.

Table S5. Hospitalization and Moderate-to-Severe Case Rates by Age Group

This table presents the total number of children covered by Maccabi Healthcare Services, hospitalizations, and moderate-to-severe cases across five pediatric age groups, along with hospitalization and moderate-to-severe case rates per 100,000 children per year. Confidence intervals (95% CI) are provided for rate estimates.

Age Group	Total Number of Children	Number of Hospitalizations	Moderate-to-Severe Cases	Hospitalization Rate per 100,000 per Year	Moderate-to-Severe Case Rate per 100,000 per Year
0-1	42,831	240	11	156.4 (95% CI: 137.8, 177.4)	7.2 (95% CI: 4.0, 12.8)
1-3	88,415	286	41	90.3 (95% CI: 80.4, 101.3)	12.9 (95% CI: 9.5, 17.6)
3-12	388,990	225	27	16.1 (95% CI: 14.2, 18.4)	1.9 (95% CI: 1.3, 2.8)
12-15	125,998	74	15	16.4 (95% CI: 13.1, 20.6)	3.3 (95% CI: 2.0, 5.5)
15-18	123,373	110	19	24.9 (95% CI: 20.6, 30.0)	4.3 (95% CI: 2.8, 6.7)
**Total**	**769,607**	**935**	**113**	**33.9 (95% CI: 31.8, 36.1)**	**4.1 (95% CI: 3.4, 4.9)**

Supplementary S5: Sensitivity Analyses

We performed 9 sensitivity analyses. First, we repeated the main analysis while adjusting for **population group** (Jewish, Ultra-Orthodox, Arab) and **socio-economic status** (Low, Medium, High) factors. The results remained largely unchanged, suggesting that these factors had minimal impact on the main findings (Table S6; n=935).

Second, we repeated the analysis after **excluding infants** (aged 0–1 year), given that this age group is often hospitalized based on precautionary clinical guidelines rather than disease severity. The exclusion had minimal impact on the results, suggesting that including infants in the main analysis might have introduced negligible bias (Table S7; n = 695).

Third, we conducted an **infant-only** analysis (Table S8; n = 240), which yielded point estimates directionally consistent with the main analysis but with wider confidence intervals, reflecting reduced statistical power due to the smaller sample size. In this subgroup, male gender was associated with higher odds of moderate-to-severe COVID-19 (OR = 8.83; 95% CI: 1.05–73.98; p = 0.045), although estimates were highly imprecise. A non-parametric permutation test comparing proportions of moderate-to-severe disease between males and females, based on random reassignment of sex labels, yielded a similar inference (p = 0.011).

Fourth, we conducted a sensitivity analysis **restricted to vaccine-eligible** children aged 5–18 years, excluding those aged 0–5 years due to negligible vaccination coverage in this younger age group. In this subgroup (n=338), unvaccinated children had higher odds of moderate-to-severe disease, and comorbidity and older age remained strongly associated with severe outcomes. Results were consistent in both standard logistic regression **and penalized logistic** regression using Firth's correction, with similar direction and magnitude of effects across models (**Tables S9-10**; n = 338).

Fifth, we repeated the main analysis while **modifying the operational definition of vaccination** status. In this model, vaccinated children were defined as those hospitalized between **14 days and 6 months** after receiving a second or third dose of the COVID-19 vaccine, instead of the original 12-month window used in the primary analysis. The results remained directionally consistent, but the association between vaccination and moderate-to-severe disease was reduced and no longer statistically significant (OR = 3.87; 95% CI: 0.84–17.76; p = 0.082; Table S11).

Sixth, we conducted a sensitivity analysis using a **broader definition of vaccination status**. In this model, vaccinated children were defined as those hospitalized between 14 days and 12 months after receiving any COVID-19 vaccine dose, **including a single dose** (rather than only a second or third dose, as in the main analysis). The results remained directionally consistent with the main model. The association between vaccination and reduced odds of moderate-to-severe disease remained statistically significant, though the estimated effect was somewhat smaller (OR = 4.62; 95% CI: 1.31–16.31; p = 0.017; Table S12).

Seventh, **univariable (unadjusted)** logistic regression analyses were conducted for each covariate included in the primary model (Table S13; n = 935), yielding crude estimates that were directionally consistent with the adjusted results.

Eighth, we repeated the main analysis using **penalized** logistic regression with Firth's correction to address potential small-sample bias due to sparse events among vaccinated children. The results were similar in direction and magnitude to the primary multivariable model, with unvaccinated children remaining at substantially higher odds of moderate-to-severe disease (Table S14; n = 935).

Ninth**,** we conducted a sensitivity analysis in which the **Delta period was analyzed separately** from earlier pre-Omicron months. This allowed us to examine whether pooling all pre-Omicron months influenced the estimated associations. The results were qualitatively similar to the main analysis, with comparable patterns for age, comorbidity, and vaccination status (Table S15; n = 935).

Overall, across all nine sensitivity analyses, the direction and magnitude of the associations were consistent with those in the main model. These findings support the robustness of our conclusions regarding the key predictors of moderate-to-severe disease among hospitalized children.

Table S6. *Multivariable-adjusted logistic results for hospitalized children (n = 935), including* ***population group*** *and* ***socioeconomic*** *status in the analysis, presenting odds ratios for moderate-to-severe disease among hospitalized patients.*

**Predictors**	**Category**	**Odds Ratios**	**CI**	**p-value**
Gender	Female	1	*Reference Level*	
	Male	1.49	0.97 – 2.27	0.069
Period	Pre-Omicron	1	*Reference Level*	
	Omicron BA.1-2	0.82	0.50 – 1.34	0.423
	Post-Omicron	0.61	0.34 – 1.11	0.106
Comorbidity	0	1	*Reference Level*	
	1	2.04	1.31 – 3.18	0.002
	"+2"	5.92	2.75 – 12.73	<0.001
Age group	0–1	1	*Reference Level*	
	1–3	2.92	1.43 – 5.96	0.003
	3–12	2.57	1.20 – 5.48	0.015
	12–15	6.07	2.55 – 14.45	<0.001
	15–18	4.97	2.14 – 11.54	<0.001
Vaccination status	Vaccinated	1	*Reference Level*	
	Unvaccinated	5.82	1.30 – 26.10	0.021
Population Group	Jewish	1	*Reference Level*	
	Arab	1.84	0.78 – 4.32	0.162
	UltraOrthodox	1.57	0.83 – 2.95	0.163
Socioeconomic Status	Low	1	*Reference Level*	
	Medium	1.19	0.62 – 2.29	0.6
	High	1.58	0.72 – 3.44	0.254

***Table S7**. Multivariable-adjusted logistic results for hospitalized children **excluding infants** (aged 1–18; n = 695), presenting odds ratios for moderate-to-severe disease among hospitalized patients.*

	**Predictors**	**Odds Ratios**	**CI**	**p-value**
Gender	Female	**1**	*Reference Level*	
	Male	1.28	0.82 – 1.99	0.272
Period	Pre-Omicron	**1**	*Reference Level*	
	Omicron BA.1-2	0.84	0.51 – 1.40	0.51
	Late Omicron era	0.64	0.34 – 1.21	0.169
comorbidity	0	**1**	*Reference Level*	
	1	1.86	1.16 – 2.98	0.01
	"+2"	6.01	2.78 – 12.99	<0.001
Age group	1-2	**1**	*Reference Level*	
	3-12	0.86	0.50 – 1.48	0.593
	12-15	2.09	1.04 – 4.21	0.039
	15-18	1.65	0.84 – 3.23	0.146
Vaccination status	vaccinated	**1**	*Reference Level*	
	Unvaccinated	6.09	1.35 – 27.39	0.019

***Table S8**. Multivariable-adjusted logistic results for hospitalized children **restricted to infants** (Aged 0–1 years; n = 240), presenting odds ratios for moderate-to-severe disease among hospitalized patients.*

Predictors	Category	Odds Ratios	CI	p-value
Gender	Female	1	*Reference Level*	
	Male	8.83	1.05 – 73.98	**0.045**
Period	Pre-Omicron	1	*Reference Level*	
	Omicron BA.1-2	0.48	0.05 – 4.43	0.514
	Late Omicron era	0.37	0.09 – 1.51	0.168
Comorbidity	No	1	*Reference Level*	
	Yes	2.07	0.54 – 7.91	0.289
Age group (months)	0-2	1	*Reference Level*	
	2-6	4.09	0.97 – 17.30	0.055
	6-12	5.69	0.86 – 37.52	0.071

Table S9. *Multivariable-adjusted logistic regression model for moderate-to-severe disease among hospitalized children aged **5–18** years (n = **338**). Odds ratios are presented. Estimates are adjusted for age group, gender, time period, comorbidity, and vaccination status.*

	**Predictors**	**Odds Ratios**	**CI**	**p-value**
**Gender**	Female	**1**	*Reference Level*	
	Male	1.16	0.61 – 2.19	0.646
**Period**	Pre-Omicron	**1**	*Reference Level*	
	Omicron BA.1-2	0.59	0.27 – 1.31	0.195
	Late Omicron era	0.62	0.24 – 1.58	0.315
**comorbidity**	0	**1**	*Reference Level*	
	1	2.46	1.23 – 4.92	0.011
	"+2"	5.52	1.97 – 15.48	0.001
**Age group**	5-12	**1**	*Reference Level*	
	12-15	2.46	1.11 – 5.49	0.027
	15-18	1.89	0.88 – 4.08	0.105
**Vaccination status**	Vaccinated	**1**	*Reference Level*	
	Unvaccinated	5.22	1.13 – 24.19	0.035

Table S10. *Multivariable-adjusted* ***penalized*** *logistic regression model (Firth) for moderate-to-severe disease among hospitalized children aged* ***5–18*** *years (n =* ***338****). Odds ratios are presented. Estimates are adjusted for age group, gender, time period, comorbidity, and vaccination status.*

	**Predictors**	**Odds Ratios**	**CI**	**p-value**
**Gender**	Female	**1**	*Reference Level*	
	Male	1.15	0.62 – 2.12	0.656
**Period**	Pre-Omicron	**1**	*Reference Level*	
	Omicron BA.1-2	0.61	0.29 – 1.31	0.206
	Late Omicron era	0.65	0.27 – 1.59	0.345
**comorbidity**	0	**1**	*Reference Level*	
	1	2.42	1.24 – 4.71	0.012
	"+2"	5.3	1.98 – 14.18	0.002
**Age group**	5-12	**1**	*Reference Level*	
	12-15	2.41	1.11 – 5.20	0.029
	15-18	1.85	0.88 – 3.89	0.108
**Vaccination status**	Vaccinated	**1**	*Reference Level*	
	Unvaccinated	4.16	1.09 – 15.96	0.021

Table S11. *Multivariable-adjusted logistic results among hospitalized children (n = 935), showing odds ratios for moderate-to-severe disease. Vaccinated children are defined as those hospitalized* ***between 14 days and 6 months*** *after receiving a second or third dose of the COVID-19 vaccine.*

	**Predictors**	**Odds Ratios**	**CI**	**p-value**
**Gender**	Female	**1**	*Reference Level*	
	Male	1.47	0.96 – 2.24	0.073
**Period**	Pre-Omicron	**1**	*Reference Level*	
	Omicron BA.1-2	0.76	0.47 – 1.23	0.27
	Late Omicron era	0.55	0.31 – 0.97	**0.039**
**Comorbidity**	0	**1**	*Reference Level*	
	1	2.03	1.30 – 3.15	**0.002**
	"+2"	5.8	2.73 – 12.31	**<0.001**
**Age group**	0–1	**1**	*Reference Level*	
	1–3	3	1.47 – 6.11	**0.002**
	3–12	2.58	1.22 – 5.46	**0.014**
	12–15	5.88	2.50 – 13.85	**<0.001**
	15–18	4.4	1.93 – 10.03	**<0.001**
**Vaccination status**	vaccinated	**1**	*Reference Level*	
	Unvaccinated	3.87	0.84 – 17.76	0.082

Table S12. *Multivariable-adjusted logistic regression results among hospitalized children (n = 935), showing odds ratios for moderate-to-severe disease. Vaccinated children are defined as those hospitalized between 14 days and 12 months after receiving* ***any*** *COVID-19 vaccine dose (including a* ***single dose****).*

	**Predictors**	**Odds Ratios**	**CI**	**p-value**
**Gender**	Female	**1**	*Reference Level*	
	Male	1.51	0.99 – 2.30	0.057
**Period**	Pre-Omicron	**1**	*Reference Level*	
	Omicron BA.1-2	0.8	0.49 – 1.30	0.374
	Post-Omicron	0.63	0.35 – 1.13	0.124
**Comorbidity**	0	**1**	*Reference Level*	
	1	1.94	1.25 – 3.03	**0.003**
	"+2"	5.69	2.67 – 12.12	**<0.001**
**Age group**	0–1	**1**	*Reference Level*	
	1–3	3.03	1.49 – 6.17	**0.002**
	3–12	2.66	1.25 – 5.64	**0.011**
	12–15	6.69	2.80 – 15.99	**<0.001**
	15–18	5.07	2.20 – 11.70	**<0.001**
**Vaccination status**	Vaccinated	**1**	*Reference Level*	
	Unvaccinated	4.62	1.31 – 16.31	**0.017**

Table S13*. *Univariable (**unadjusted**) logistic regression models for moderate-to-severe disease among hospitalized children (n = 935), presenting odds ratios for moderate-to-severe disease among hospitalized patients.

	**Predictors**	**Odds Ratios**	**CI**	**p-value**
**Gender**	Female	**1**	*Reference Level*	
	Male	1.47	0.98–2.22	0.064
**Period**	Pre-Omicron	**1**	*Reference Level*	
	Omicron BA.1-2	0.81	0.52–1.26	0.349
	Late Omicron era	0.52	0.29–0.87	0.017
**Comorbidity**	0	**1**	*Reference Level*	
	1	1.81	1.19–2.76	0.0055
	"+2"	6.11	2.91–12.48	**<0.001**
**Age group**	0–1	**1**	*Reference Level*	
	1–3	3.48	1.81–7.27	**<0.001**
	3–12	2.84	1.41–6.11	0.005
	12–15	5.29	2.33–12.41	**<0.001**
	15–18	4.35	2.02–9.79	**<0.001**
**Vaccination status**	Vaccinated	**1**	*Reference Level*	
	Unvaccinated	3.52	1.07–21.71	0.0841

Table S14. *Multivariable-adjusted* ***penalized*** *logistic regression model (Firth) for* ***moderate-to-severe*** *disease among hospitalized children (n = 935), presenting adjusted odds ratios by age group, gender, time period, comorbidity, and vaccination status.*

**Predictors**	**Category**	**Odds Ratios**	**CI**	**p-value**
Gender	Female	1	*Reference Level*	
	Male	1.48	0.98 – 2.24	0.063
Period	Pre-Omicron	1	*Reference Level*	
	Omicron BA.1-2	0.8	0.50 – 1.28	0.358
	Late Omicron era	0.62	0.35 – 1.10	0.098
Comorbidity	0	1	*Reference Level*	
	1	1.96	1.27 – 3.03	0.003
	"+2"	5.75	2.75 – 12.01	<0.001
Age group	0–1	1	*Reference Level*	
	1–3	2.91	1.46 – 5.82	0.001
	3–12	2.56	1.23 – 5.31	0.01
	12–15	6.17	2.67 – 14.26	<0.001
	15–18	4.84	2.15 – 10.88	<0.001
Vaccination status	Vaccinated	1	*Reference Level*	
	Unvaccinated	4.77	1.25 – 18.17	0.008

Table S15. *Multivariable-adjusted logistic regression model for moderate-to-severe disease among hospitalized children (n = 935), presenting adjusted odds ratios by age group, gender, pandemic period, comorbidity, and vaccination status. In this analysis, the* ***Delta period*** *was modeled separately from earlier pre-Omicron periods, with "Pre-Delta" defined as waves 1 to 3, see Table A.*

Predictors	Category	Odds Ratios	CI	p-value
Gender	Female	1	*Reference Level*	
	Male	1.49	0.98 – 2.28	0.063
Period	Pre-Delta	1	*Reference Level*	
	Delta	1.84	0.96 – 3.53	0.358
	Omicron BA.1-2	0.99	0.57 – 1.72	0.098
	Late Omicron era	0.77	0.41 – 1.45	0.418
Comorbidity	0	1	*Reference Level*	
	1	1.96	1.26 – 3.06	0.003
	"+2"	5.72	2.67 – 12.25	<0.001
Age group	0–1	1	*Reference Level*	
	1–3	3.29	1.60 – 6.75	0.001
	3–12	2.61	1.23 – 5.54	0.013
	12–15	6.61	2.78 – 15.69	<0.001
	15–18	5.77	2.47 – 13.50	<0.001
Vaccination status	Vaccinated	1	*Reference Level*	
	Unvaccinated	6.58	1.44 – 29.95	0.015

Supplementary S6: Analysis Using a Stricter Severity Outcome Definition

Table S16. *Distribution of COVID-19 severity by vaccination status among all hospitalized children aged 0–18 years (n = 935), under non-strict and* ***strict*** *outcome definitions.*
Non-strict outcome is defined as mild vs moderate/severe/critical/death; strict outcome is defined as mild/moderate vs severe/critical/death.

Outcome Definition	Severity Type	Vaccinated	Unvaccinated
Non-strict Outcome	Mild	49	773
	Moderate/Severe/Critical/Death	2	111
Strict Outcome	Mild/Moderate	51	828
	Severe/Critical/Death	0	56

Table S17. *Same as Table S16, restricted to hospitalized vaccine-eligible children aged 5–18 years (n = 338).*

Outcome Definition	Severity Type	Vaccinated	Unvaccinated
Non-strict Outcome	Mild	49	238
	Moderate/Severe/Critical/Death	2	49
Strict Outcome	Mild/Moderate	51	259
	Severe/Critical/Death	0	28

***Table S18**. Absolute risks and risk differences for COVID-19 severity by vaccination status among hospitalized children aged 0–18 years (n = 935).*

Outcome Definition	Group	Events / N	Absolute risk (95% CI)
Non-strict outcome	Vaccinated	2 of 51	3.9% (95% CI: 1.1%-13.2%)
	Unvaccinated	111 of 884	12.6% (95% CI: 10.5%-14.9%)
	Risk difference	--------	-8.6% (95% CI: -13.8%-2.7%)
Strict outcome	Vaccinated	0 of 51	0.0% (95% CI: 0.0%-7.0%)
	Unvaccinated	56 of 879	6.4% (95% CI: 4.9%-8.2%)
	Risk difference	-------	-6.4% (95% CI: -8.2%-2.1%)

***Table S19**. Same as Table S18, restricted to hospitalized vaccine-eligible children aged 5–18 years (n = 338).*

Outcome Definition	Group	Events / N	Absolute risk (95% CI)
Non-strict outcome	Vaccinated	2 of 51	3.9% (95% CI: 1.1%-13.2%)
	Unvaccinated	49 of 287	17.1% (95% CI: 13.2%-21.9%)
	Risk difference	--------	-13.2% (95% CI: -20.8%-0.1%)
Strict outcome	Vaccinated	0 of 51	0.0% (95% CI: 0.0%-7.0%)
	Unvaccinated	28 of 287	9.8% (95% CI: 6.8%-13.7%)
	Risk difference	-------	-9.8% (95% CI: -13.7%-0.2%)

Table S20. *Multivariable-adjusted* ***standard*** *logistic regression model for* ***strict*** *severe outcomes (severe/critical illness or death vs mild/moderate disease) among hospitalized children (n = 935). Odds ratios are adjusted for age group, gender, time period, comorbidity, and vaccination status. No severe events occurred among vaccinated children.*

**Predictors**	**Category**	**Odds Ratios**	**CI**	**p-value**
Gender	Female	1	*Reference Level*	
	Male	1	0.57 – 1.75	1
Period	Pre-Omicron	1	*Reference Level*	
	Omicron BA.1-2	0.98	0.50 – 1.93	0.949
	Late Omicron era	1.15	0.54 – 2.44	0.714
Comorbidity	0	1	*Reference Level*	
	1	2.11	1.14 – 3.88	0.017
	"+2"	5.91	2.35 – 14.87	<0.001
Age group	0–1	1	*Reference Level*	
	1–3	5.7	1.64 – 19.79	0.006
	3–12	4.93	1.35 – 18.02	0.016
	12–15	15.29	3.90 – 59.94	<0.001
	15–18	11.18	2.86 – 43.74	0.001
Vaccination status	Vaccinated	1	*Reference Level*	
	Unvaccinated	16028074.58	0.00 – Inf	0.985

Table S21. *Multivariable-adjusted **penalized** logistic regression model (Firth correction) for **strict** severe outcomes (severe/critical illness or death vs mild/moderate disease) among hospitalized children (n = 935). Odds ratios are adjusted for age group, gender, time period, comorbidity burden, and vaccination status.*

Predictors	Category	Odds Ratios	CI	p-value
Gender	Female	1	*Reference Level*	
	Male	1	0.58 – 1.70	0.989
Period	Pre-Omicron	1	*Reference Level*	
	Omicron BA.1-2	0.98	0.51 – 1.88	0.956
	Post-Pandemic	1.16	0.57 – 2.38	0.689
Comorbidity	0	1	*Reference Level*	
	1	2.08	1.16 – 3.73	0.017
	"+2"	5.83	2.43 – 14.03	<0.001
Age group	0–1	1	*Reference Level*	
	1–3	4.99	1.59 – 15.62	0.002
	3–12	4.38	1.33 – 14.43	0.009
	12–15	13.48	3.82 – 47.52	<0.001
	15–18	9.86	2.80 – 34.66	<0.001
Vaccination status	Vaccinated	1	*Reference Level*	
	Unvaccinated	15.15	0.98 – 233.98	0.004

Supplementary S7: Distribution and Classification of Comorbidities

Table S22. *Distribution of specific comorbidities among hospitalized children (patients may have more than one condition).*

Comorbidity	n
Chronic respiratory disease (asthma)	320
Cardiovascular disease	43
Oncologic disease (current or past)	32
Diabetes mellitus	12
Medically complex/homebound	11
Inflammatory bowel disease	5
Hypertension	2
Chronic kidney disease/dialysis	1
**Total**	**426**

Table S23. *Distribution of mutually exclusive comorbidity categories among children with at least one underlying condition*

Comorbidity	n
Asthma / respiratory only	290
Medically complex/immunocompromised / oncology	49
Cardio / metabolic	45
**Total**	**384**

Supplementary S8: Primary versus Incidental COVID-19 Hospitalizations by Pandemic Period

Table S24. *Distribution of pediatric hospitalizations by COVID-19 admission type and pandemic period. The number and proportion of pediatric hospitalizations classified as primary COVID-19 admissions (hospitalized due to COVID-19) and incidental SARS-CoV-2–positive admissions (hospitalized for other reasons with an incidental positive test), stratified by pandemic period. Percentages are calculated within each period.*

COVID-19 Admission Type	Pre-Omicron	Omicron BA.1–2	Late Omicron era
Primary	369 (81.5%)	320 (83.6%)	246 (88.8%)
Incidental	84 (18.5%)	63 (16.4%)	31 (11.2%)

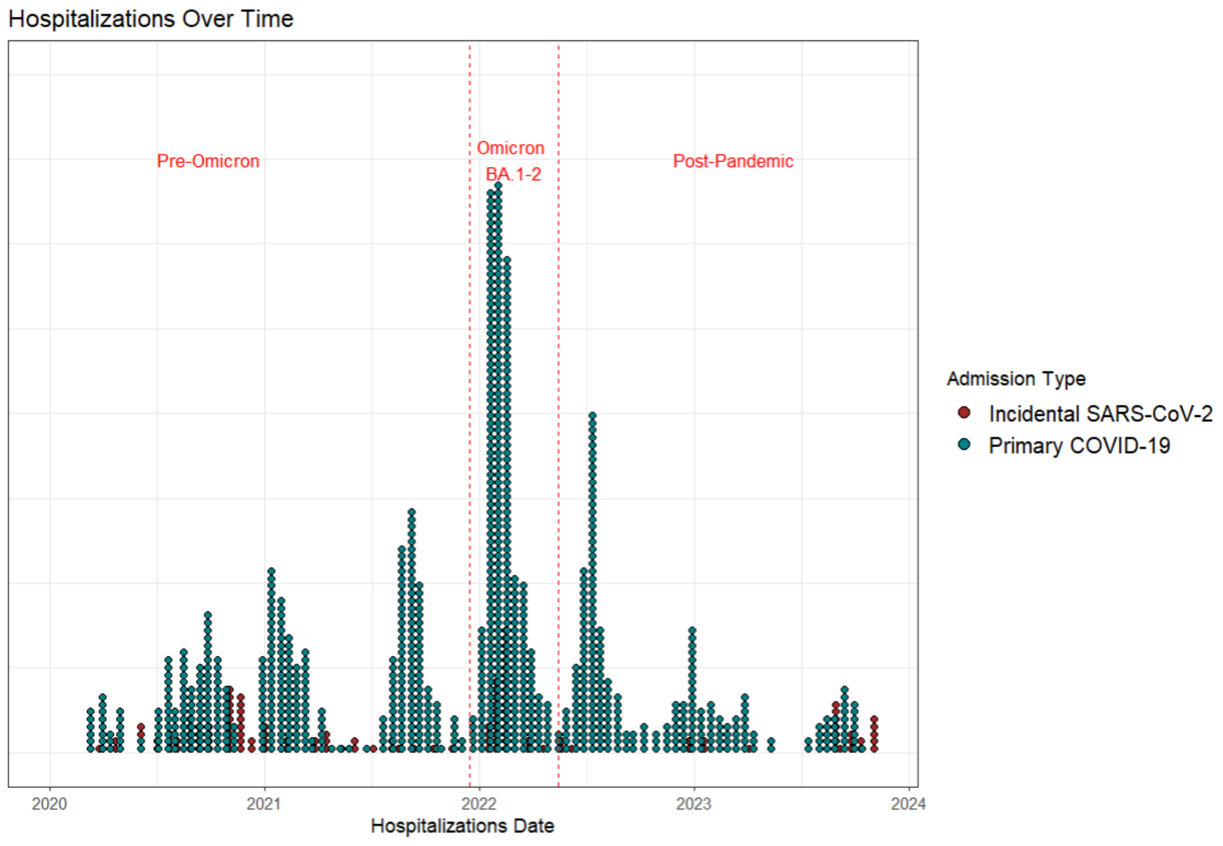

Figure S4. *Hospitalizations over time by admission type. Hospitalization dates are grouped into 14-day bins. Each dot represents a single hospitalization, and dots are stacked vertically when multiple hospitalizations occurred within the same window. Colors indicate primary COVID-19 versus incidental SARS-CoV-2 admissions.*

Supplementary S9: Clinical Severity of COVID-19 Hospitalizations

Table S25. *Overall distribution of clinical severity among COVID-19 hospitalizations. Percentages are calculated out of all children hospitalized* ***due to*** *COVID-19 in the analytic cohort (n = 935).*

Severity	Count (percent)
Mild	822 (87.9%)
Moderate	57 (6.1%)
Severe	44 (4.7%)
Critical	11 (1.2%)
Death	1 (0.1%)
**Total**	**935 (100%)**

Table S26. *Distribution of clinical severity among COVID-19 hospitalizations by pandemic period. Percentages are calculated out of all children hospitalized* ***due to*** *COVID-19 in the analytic cohort (n = 935).*

Severity	Pre-Omicron	Omicron BA.1–2	Late Omicron era
Mild	315 (85.4%)	281 (87.8%)	226 (91.9%)
Moderate	30 (8.1%)	20 (6.2%)	7 (2.8%)
Severe	18 (4.9%)	15 (4.7%)	11 (4.5%)
Critical	6 (1.6%)	4 (1.2%)	1 (0.4%)
Death	0	0	1 (0.4%)
**Total**	**369 (100%)**	**320 (100%)**	**246 (100%)**

References

[1] חברות בקופת חולים 2023 (מס. 323) - סקרים תקופתיים | ביטוח לאומי. www.btl.gov.il n.d. http://www.btl.gov.il:80/Publications/survey/Pages/skira20230912-9996.aspx (accessed June 18, 2025).

[2] חברות בקופת חולים, 2020 - סקרים תקופתיים | ביטוח לאומי. www.btl.gov.il n.d. http://www.btl.gov.il:80/Publications/survey/Pages/seker_320.aspx (accessed June 18, 2025).
